# Notch Signaling in the Bone Marrow Lymphopoietic Niche

**DOI:** 10.3389/fimmu.2021.723055

**Published:** 2021-07-28

**Authors:** Kilian Sottoriva, Kostandin V. Pajcini

**Affiliations:** Department of Pharmacology and Regenerative Medicine, University of Illinois at Chicago College of Medicine, Chicago, IL, United States

**Keywords:** lymphopoiesis, hematopoieisis, Notch signaling, T cell development, bone marrow niches

## Abstract

Lifelong mammalian hematopoiesis requires continuous generation of mature blood cells that originate from Hematopoietic Stem and Progenitor Cells (HSPCs) situated in the post-natal Bone Marrow (BM). The BM microenvironment is inherently complex and extensive studies have been devoted to identifying the niche that maintains HSPC homeostasis and supports hematopoietic potential. The Notch signaling pathway is required for the emergence of the definitive Hematopoietic Stem Cell (HSC) during embryonic development, but its role in BM HSC homeostasis is convoluted. Recent work has begun to explore novel roles for the Notch signaling pathway in downstream progenitor populations. In this review, we will focus an important role for Notch signaling in the establishment of a T cell primed sub-population of Common Lymphoid Progenitors (CLPs). Given that its activation mechanism relies primarily on cell-to-cell contact, Notch signaling is an ideal means to investigate and define a novel BM lymphopoietic niche. We will discuss how new genetic model systems indicate a pre-thymic, BM-specific role for Notch activation in early T cell development and what this means to the paradigm of lymphoid lineage commitment. Lastly, we will examine how leukemic T-cell acute lymphoblastic leukemia (T-ALL) blasts take advantage of Notch and downstream lymphoid signals in the pathological BM niche.

## Introduction

Notch signaling is a highly conserved pathway activated through cell-to-cell, ligand-receptor interactions. There are five Notch ligands in mammals: Delta like (Dll) 1, 3 and 4 and Jagged (Jag) 1 and 2 which are presented on the surface of multiple cells and tissues (1). When the ligand interacts with one of the 4 mammalian Notch receptors, (Notch1-4) a series of proteolytic cleavages releases the Notch receptor from the plasma membrane (2). Subsequently, the intracellular Notch (ICN) domain translocates to the nucleus, where it binds to Recombining Binding Protein Suppressor of Hairless (RBP-J) and co-activator Mastermind-like (MAML) (2, 3). Ultimately, it is this tri-molecular complex that binds to enhancer and promoter elements to initiate transcriptional activation of target genes. Along with Wtn, Hedgehog, and Bone Morphogenic Peptide/TGF- β, Notch signaling is one of the fundamental pathways essential for mammalian embryogenesis (4). Notch signaling plays a multitude of roles in the differentiation, proliferation, self-renewal, and survival in diverse cell types across many tissues (5). Particularly well studied are the roles of Notch1 in somite segmentation (6, 7), in angiogenesis and vascular development (8, 9), and the emergence of the definitive hematopoietic stem cell (HSC) in the aorta-gonad-mesonephros (10, 11). In the post-natal murine BM, HSC cell-autonomous and non-cell-autonomous Notch signaling has been implicated in several contexts including aging, regeneration, and mobilization, reviewed in these studies (12–15). Though loss-of-function studies in adult mice do not support a requirement for HSC cell-autonomous Notch activation during homeostasis (16, 17) and in one more recent study in regenerative hematopoiesis (18), Notch signaling has been implicated in the development of several different blood lineages, including megakaryocytes (19), NK cells (20), and erythrocytes (21). Yet, it is the role of Notch signaling in the cell fate determination of the T cell lineage that remains as the archetypic function of the pathway in adult hematopoiesis (22, 23).

The developmental progression from the BM HSC to the production of functional peripheral T cells is physiologically continuous but can be delineated using surface markers and expression of key transcriptional regulators. In both mouse and human, BM lymphoid progenitors give rise to thymic precursors, which progress through well-defined developmental stages in the thymus to become naïve T cells (24). Progression through distinct stages of thymic T cell development requires the careful coordination of several lineage regulatory transcription factors, including: Ikaros, Gfi1, Myb, Runx family proteins, E2A, HEB, TCF1, GATA3, Bcl11b, LEF1, and of course Notch1 (25). In the thymus, the roles of Notch signaling have been well studied. After homing to the thymus, early progenitors activate Notch signaling, which is required for thymocyte development (26–28). Notch is implicated in a variety of functions such as inhibition of progenitor apoptosis, induction of T cell lineage master regulators *Gata3*, *Tcf7*, and *Bcl11b*, as well as activation of genes involved in functional T cell receptor (TCR) production such as *Ptcrα* (29–31). Notch signaling becomes dispensable for T cell differentiation after β-selection occurs, at which point subsequent development is dependent on signals from the pre-TCR complex (29, 32). The main receptor expressed by thymocytes is Notch1, while the major Notch ligand expressed by cortical Thymic Epithelial Cells (TECs) is Delta-like 4 (Dll4) (30, 33). While the role of Notch in T cell development in undeniable, the temporal and spatial aspects of the first requirement for Notch in driving T cell fate have not been fully established. Recently, several findings have begun to address this issue by suggesting that pre-thymic Notch signals influence the ability of primitive BM lymphoid progenitors to produce thymus-seeding cells (18, 34, 35). Here, we will review the work which encompasses our current understanding of the BM populations that give rise to thymic progenitors, and the role of Notch signaling as a niche component in driving this process. Under this new paradigm of pre-thymic Notch activation, we will then examine the pathological Notch-dependent mechanisms of the lymphoid niche in the leukemic BM environment.

## BM Thymocyte Progenitors

**Figure 1:**
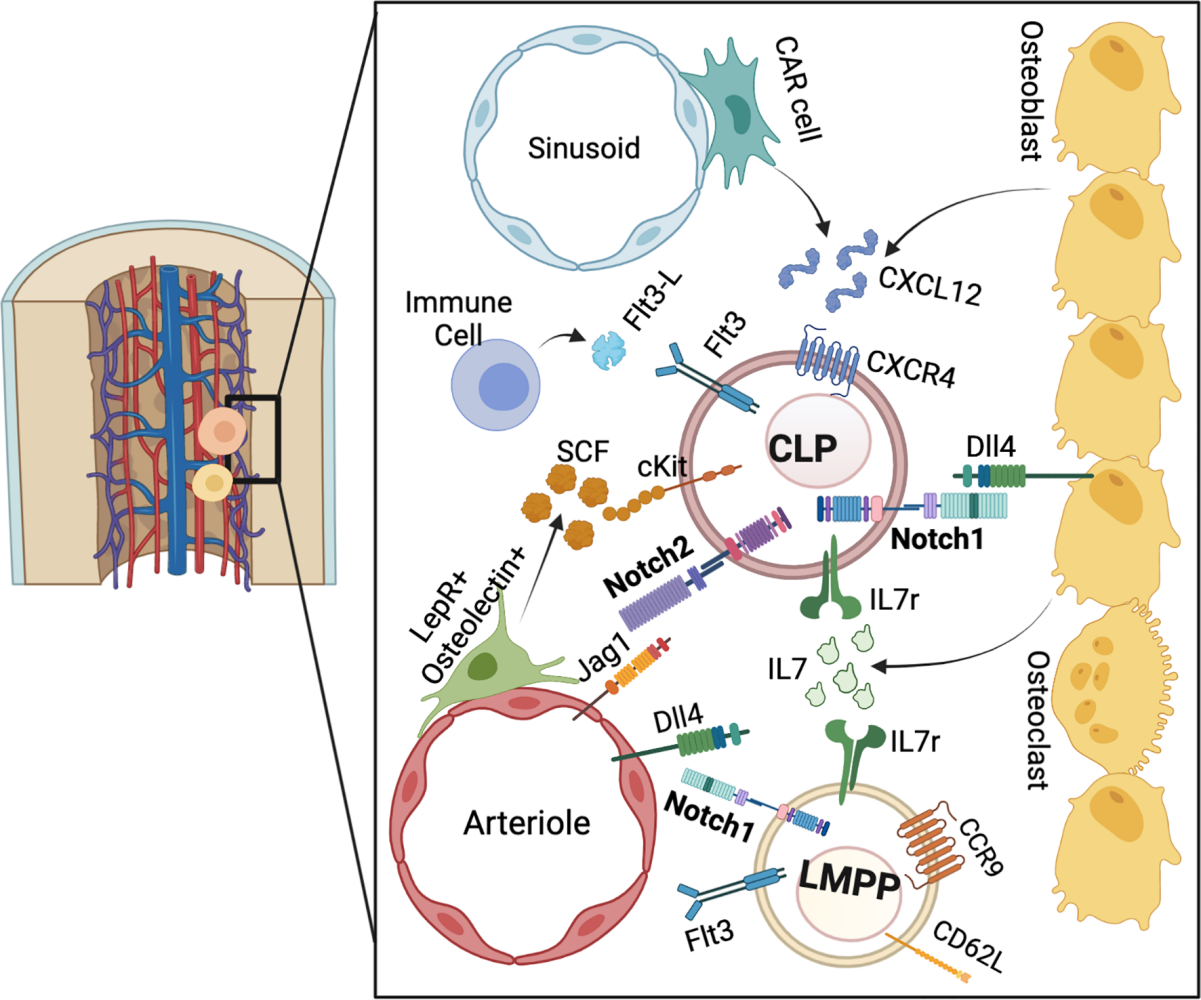
The BM niche for pre-thymic T cell progenitor development. LMPPs and CLPs reside in the endosteal niche. Notch, IL7r, and CXCR4 ligands are derived from the osteoblastic and stromal niche, while SCF is provided from peri-arteriolar cells. Flt3L is provided by mature immune cells. Overall, these signaling pathways converge to stimulate lymphoid progenitors to the T cell lineage.

In adult mammals, the hematopoietic system is maintained *via* the production of functional blood cells and hematopoietic progenitors by self-renewing HSCs in the BM (36). The BM microenvironment is composed of osteoprogenitors, stromal cells, endothelial cells, and multiple hematopoietic cell types (**Figure 1**) (37, 38). At the apex of BM hematopoiesis is the HSC, which is defined best by its self-renewal and functional capacity to produce all the lineages of blood rather than by a specific set of markers. Even so, for isolation purposes the HSC has been classified by surface markers as Lin^-^cKit^+^Sca1^+^CD150^+^CD48^-^ (39), by the presence of efflux pumps (40) and by the expression of intracellular proteins including Hoxb5 and α-catulin (41, 42). Next in the hematopoietic hierarchy are the HSPCs, which in murine hematopoiesis are generally classified by the combination of Lin^-^cKit^+^Sca1^+^ and become increasingly lineage committed. This differentiation potential arises at the expense of the capacity to self-renew (43, 44). As the HSPCs gain lineage specific potential, they begins to express surface proteins which have been used to define specific progenitor populations in the BM, termed Multipotent Progenitor Populations (MPP) (45–47).

In the case of early BM lymphopoiesis, several progenitor populations have been described. Cells within the HSPC pool which express the tyrosine kinase receptor Fms-like tyrosine kinase 3 (Flt3) have been labeled as lymphoid-primed MPPs (LMPP), also termed MPP4 (45, 48–50). LMPP lineage output is functionally distinct from myeloid biased MPP2 (Flt3^-^CD48^+^CD150^+^) and MPP3 (Flt3^-^CD48^+^CD150^-^) populations as determined *via* murine transplantation experiments (50). LMPPs were shown to have equivalent B and T cell potential, retain some granulocyte and monocyte potential, but lack the ability to produce erythroid and megakaryocyte lineages (49, 51). LMPPs can be further segregated into lymphoid biased cells through expression of a selection of surface proteins. The Interleukin 7 receptor (IL7r), which is required for lymphoid development, is expressed on a subset of LMPPs which efficiently generate T cells and innate lymphoid cells in a murine transplantation setting (52, 53). L-selectin (CD62L) is involved in the trafficking of naïve lymphocytes to peripheral lymphoid organs by binding to a selection of different glycan residues and can be used to specify T lineage progenitors in the BM (54–57). Expression of CD62L separates LMPPs which have transient B cell potential and can yield rapid thymocyte production, but lack the ability to produce cells of myeloid lineages (58). Furthermore, CD62L upregulation has been shown to be an early event in the lymphoid priming of human BM progenitors (59).

Additionally, Vascular Cell Adhesion Molecule 1 (VCAM1) and Flt3 expression can be used to segregate MPPs with combined lymphoid/myeloid (Flt3^hi^VCAM1^+^), erythroid (Flt3^lo^VCAM1^+^), or B and T cell potential (Flt3^hi^VCAM1^-^) (60). VCAM1 is a cell adhesion molecule with roles in vascular adhesion and transendothelial migration of leukocytes (61, 62). Originally identified on the surface of endothelial cells (63, 64), VCAM1 has since been found to be expressed on the surface of multiple cell types including hematopoietic progenitors, macrophages, and BM fibroblasts (65). It is through ligand binding, specifically the α4β1 integrin (CD49d/CD29) and α4β7 integrins expressed on the surface of leukocytes, that VCAM1 mediates adhesion and transmigration of T cells and macrophages (61, 66). The VCAM1^-^ LMPP population in the BM homogeneously expresses Flt3, and expression of C-C chemokine Receptor type 9 (CCR9), which also is suggested to play a major role in the recruitment of BM derived cells to the thymus, further delineates a subset of T cell progenitors (67–69). Taken together, MPPs expressing Flt3, IL7r, CD62L, CCR9 and lacking VCAM1 appear to be the main HSPC component contributing to B and T cell development.

Downstream of the LMPP population, the Common Lymphoid Progenitor (CLP), which in murine hematopoiesis is isolated by surface markers Lin^-^cKit^Lo^Sca1^Lo^Flt3^+^IL7r^+^, represents a canonical branching point between myeloid and lymphoid development and is restricted for lineage production of Natural Killer (NK), B cell and T cell development (70). IL7 signaling is critically involved in BM B cell and thymic T cell lymphopoiesis, and the CLP population is defined by IL7r expression (70–72). Surface expression of Lymphocyte Antigen 6 Family Member D (Ly6D) can be used to divide the CLP population into those with T cell biased potential (Ly6D^-^) and B cell biased potential (Ly6D^+^) (73–75). Downstream of the CLP in bone marrow NK development is the Pre-Natural Killer Progenitor (Pre-NKP) and Refined Natural Killer Progenitor (rNKP) (76). In B cell development, the CLP differentiates into a series of BM sub-populations traditionally referred to as the Hardy Fractions (43), of which Fraction A (B220^+^CD43^+^CD24^−^BP-1^-^) is immediately downstream of the CLP (77–79). The next well-defined downstream T cell lineage progenitor of the CLP is the early T lineage progenitor (ETP), which is the earliest T cell progenitor in the thymus (80, 81).

While both LMPPs and CLPs possess T cell lineage potential, efforts to determine the exact BM progenitor population which is the Thymic Seeding Progenitor (TSP) have yielded conflicting reports. Although transplantation of CLPs yields thymus engraftment and thymopoiesis, Ikaros-deficient mice have been shown to have thymic ETPs without a detectable CLP population in the BM (82, 83). Additionally, IL7r^+^ LMPPs can generate thymocytes in a CLP-independent manner post-transplant (53). A potential resolution for this issue has been proposed during pre-natal thymopoiesis, where TSPs are produced in two separate waves, the first of which resembles CLPs and a second resembling LMPPs (84). However, a caveat to these findings is the use of transplantation to determine functional kinetics of TSP generation, which requires removal of these BM resident populations and injection into a recipient’s bloodstream. Thus, while TSP generation is determined *in vivo*, the path of the populations in question from the BM to the bloodstream and finally the thymus is in the form of a transplant and does not necessarily mimic natural BM egress and thymic homing. This a principle commonly seen in HSC studies, where it has been recognized that transplantation leads to oligoclonal dominance that does not reflect unperturbed hematopoiesis (85, 86). In order to more accurately determine the BM source for TSP generation, additional methods such as *in vivo* lineage tracing and single cell RNA sequencing should be applied, such as in recent work which unveiled direct production of megakaryocyte progenitors (MkP) from long term HCSs (LT-HSCs) (87). Thus, while the exact BM population for TSP generation has yet to be specifically determined, both the LMPP and CLP populations remain viable sources.

## Pre-Thymic Notch Signaling in Lymphopoiesis

Notch signaling is essential for T cell development as shown by the seminal loss-of-function studies by Radtke F et al. (28). However, similar loss of function experiments that deleted the DNA binding member of the trimolecular complex RBPJ (88–90) as well as pan-Notch inhibition with a Mastermind truncation named dominant-negative Mastermind (dnMAML) (17) all indicated that loss of Notch signaling in the BM HSC population had no effect on HSC homeostasis in adult mice. However, as we have described above, several stages of progenitor differentiation occur between the HSC in the BM and the emigrating TPS. The critical temporal question is whether Notch signaling is activated and required for development of the LMPP, CLP or the ETP. Early insight into the role of Notch in BM T lineage lymphopoiesis can be found in studies which showed that the CLP population expresses Notch1 at the mRNA level, and that Notch1 deficient CLP cells erroneously differentiate into B cells in the thymus (91, 92). BM cells transduced with dnMAML failed to produce ETP cells post-transplant, once again hinting at a pre-thymic role for Notch in ETP generation (26). It was further observed that CCR9+ T cell biased MPPs have the potential to activate Notch signaling (68). To determine the expression of individual Notch receptors in HSPC populations, an *in vivo* lineage tracing system has been developed. Cre-recombinase was “knocked into” the individual loci for Notch1-4, which allowed for determination of receptor expression using a fluorescent Cre-reporter mouse strain. This system revealed Notch1 expression in LMPPs, Notch1 and Notch2 expression in CLPs, and an absence of Notch3 or Notch4 in either population (21).

Abrogation of Notch signaling in the BM through inhibition or genetic deletion of Notch receptors or ligands has indicated a role for Notch-dependent T cell progenitor development in the BM. Injection of Notch ligand Dll4 neutralizing antibodies, which have been shown to block ligand specific signaling, leads to a decrease in the BM CLP population (93, 94). Consistently, deletion of either Notch ligand Dll4 or Mindbomb (Mib), which is involved in Notch ligand endocytosis, in Osteocalcin (Ocn) expressing bone cells led to a significant decrease in the CLP population (34, 95). Similar results were obtained when either RBP-J or GDP-fucose protein O-fucosyltransferase 1 (POFUT1) were deleted from BM hematopoietic progenitors (34, 96). The CLP defect observed after conditional deletion of osteoblastic Dll4 underscores the potential existence of an osteoblastic niche for Notch-dependent priming of BM lymphoid progenitors (**Figure 1**). Indeed, it has been shown that C-X-C Motif Chemokine Ligand 12 (Cxcl12) derived from osteoblasts, and not endothelial or hematopoietic cells, is required for CLP and LMPP maintenance in the BM niche (97). The osteoblastic niche has also been implicated in BM B cell progenitor development, through stimulation of HSCs towards the lymphoid lineage *via* G_s_α dependent osteoblast IL7 production (98–100). Peri-arteriolar LEPR^+^Osteolectin^+^ cells have also been shown to stimulate CLP development through secretion of SCF (**Figure 1**) (101). This osteoblast-derived SCF secretion decreases in aged mice which have an imbalance in blood lineage output with a propensity toward myeloid populations (101, 102).

While there is mounting evidence supporting the osteoblastic microenvironment as a lymphoid sub-niche in the BM, there are also reports that implicate different niche cells in the priming of lymphoid progenitors. For example, endothelial cells which express high levels of Notch ligands Dll4 and Jag1 (103) have been suggested as an alternative niche for lymphoid progenitor development. Deletion of endothelial expression of Dll4, but not Dll1, leads to a decrease in the frequency of CLP cells, with no effect on the LMPP in the BM (104). However, a direct contribution of endothelial Notch ligand to CLP Notch receptor activation was not shown, and the potential mechanism of CLP depletion was myeloid skewing of upstream HSCs. Additionally, conditional deletion of Cxcl12 in endothelial cells lead to specific depletion of HSCs in the BM, not lymphoid progenitors (97). Furthermore, deletion of SCF derived from peri-arteriolar LEPR+ cells, and not arteriolar or sinusoidal endothelial cells, depleted CLP cells in the BM (101, 105, 106). Taken together these experiments do not support a direct contribution of endothelial derived factors in lymphoid progenitor maintenance during steady-state hematopoiesis in the adult mouse bone marrow.

In most circumstances, the proposed endothelial niche, be it sinusoidal or peri-arterial (101), has been shown to sustain stemness and support self-renewal of HSCs or HSPCs, which by virtue of their hierarchical position in BM hematopoiesis yield more downstream progenitors including CLPs (107, 108). This is evident in several experiments where regenerating or expanding endothelial compartments produce more HSCs and by connection more lineage specific progenitors (109–112). Because Notch signaling is essential for endothelial growth and regeneration, and because the endothelium is a primary niche for HSCs, the effects observed in the CLP compartment could be attributed to an increase in the general abundance in HSC numbers. Disassociation of the intrinsic role of Notch signaling in arterial cell fate and endothelial function must first be shown to determine if endothelial cells represent the key components of the BM lymphopoietic niche.

Hematopoietic derived signals have also been shown to play a role in CLP homeostasis. Flt3 and Flt3-Ligand (Flt3-L) regulate both myeloid and lymphoid hematopoiesis, and Flt3-L knock-out mice have a severe defect in CLP generation (113, 114). Interestingly, Flt3-L has been shown to be produced in the BM by immune cell populations, including CD4^+^ memory T cells, rather than stromal, endothelial, or osteoblastic niche cells (115, 116). Although a role for Notch in regulation of Flt3 in homeostatic lymphopoiesis has yet to be established, canonical Notch target Hes1 transcriptionally represses Flt3 expression in Acute Myeloid Leukemia (AML) (117). Additionally, lymphoma/leukemia-Related Factor (LRF), which plays a role in erythroid and late lymphoid lineage decisions, downregulates Dll4 in BM erythroblasts, thus preventing a Notch1 dependent increase in CLP generation at the expense of the HSC pool (118–120).

Confirmation of the existence of the lymphopoietic sub-niche in the BM has been supported by other studies that have shown a cell-intrinsic role for Notch receptor activation in BM lymphoid development. Hematopoietic LRF expression promotes proper B cell development through suppression of Notch signals in CLPs, and hematopoietic deletion of LRF leads to enhanced Notch activity and extra-thymic CD4^+^CD8^+^ T cell generation in the BM (121). Hypomorphic Notch signaling achieved by deletion of the Notch1 Transcriptional Activation Domain (TAD) showed a significant decrease in CLP abundance in the BM (18, 122). Furthermore, an inducible RBPj on/off genetic mouse model has confirmed a role of pre-thymic Notch signaling. Specifically, Notch signaling through RBPj is involved in CD62L+ LMPP generation, with no effect in the T cell primed Ly6D- CLP (35). Collectively, these findings confirm a cell-intrinsic role for Notch signaling in pre-thymic T cell progenitor development in the BM microenvironment.

## Mechanisms of Notch in CLP Development

The activation of the Notch receptor is only the first step in the signaling pathway that eventually leads to transcriptional activation of target genes. While a direct role for Notch signaling in pre-thymic progenitor development is evident, the cell intrinsic mechanisms that prime T cell development downstream of Notch in the BM are unknown. A possible mechanism involves the regulation of receptors that are important for cellular migration and tissue retention. Recently, a genetic mouse model for inducible deletion and subsequent inducible expression of RBPj *in vivo* has been developed (35). In this model, floxed *Rbpj* can be conditionally deleted through an inducible Vav-Cre transgene, while a tetracycline responsive element-controlled hemagglutinin (HA)-tagged RBPJ transgene can be induced *via* doxycycline (Dox) injection. RNA sequencing of LMPP cells isolated from Rbpj^f/+^ control, RBPj knock-out, and Dox induced RBPj-HA expressing mice suggested that PSGL1, CCR7, and CCR9 are regulated by Notch signaling in the CD62L+ LMPP population (35). Additionally, deletion of Dll4 in the osteoblastic niche lead to a decrease in CLP cells expressing CCR7 and P-selectin glycoprotein ligand-1 (PSGL1) (34). PSGL1, CCR7 and CCR9 are all involved in the recruitment and migration of BM derived progenitor cells to the thymus (69, 123, 124). However, Notch signaling has also been shown to directly repress CCR9 expression in fetal liver derived T cell progenitors produced *via* co-culture with stromal cells expressing Dll1 (125). The caveat of fetal progenitor acclimation to *ex vivo* co-culture conditions could account for these contrary results. Overall, these observations highlight the possibility that Notch activation in BM lymphoid progenitors prepares cells for thymic migration through induction of genes involved in thymic homing.

Another chemokine pathway involved in BM hematopoiesis is CXCR4/CXCL12, which regulates migration, survival, and quiescence of various progenitor populations (126–130). CXCL12 is expressed by several cell types in the BM, including endothelial cells, osteoblasts, stromal cells, and hematopoietic cells (131). Interestingly, stromal CXCL12 production and HSC release from the BM have been shown to be influenced by circadian neural release of noradrenaline, which activates AdrB3 receptor on Nestin+ osteoprogenitors (132, 133). Although migration of mature leukocyte populations in the BM is regulated in part by circadian rhythms, a direct role for circadian influences on BM lymphoid progenitor biology has yet to be established (134). In humans, there is evidence that MCAM^+^CD146^+^ subendothelial stromal cells express CXCL12 (135). Hematopoietic deletion of CXCR4 results in a reduction of the BM stem cell pool. Specifically affected are HSCs in close contact with CXCL12-abundant reticular cells which surround sinusoidal endothelial cells in the BM (136). CXCR4 has also been shown to regulate the integrity of the vascular barrier in the BM, which further modulates hematopoietic trafficking (137). Work in multiple cell types has revealed dynamic regulation of CXCR4 by the Notch pathway in both mouse and human mesenchymal and endothelial cells (138–142). In the BM, Notch2 has been shown to directly activate CXCR4 expression in HSPCs, while stromal production of CXCL12 has been shown to play a role in CLP maintenance (143, 144). It should be noted, however, that blockade with a Notch2 specific antibody yields only a modest reduction of the CLP population compared to a 30% decrease with antibody blockade of Notch1 (144). Furthermore, mice expressing CXCR4 mutations derived from patients with Warts, Hypogammaglobulinemia, Infections, and Myelokathexis (WHIM) syndrome, which prevent receptor internalization and desensitization, have decreased LMPP and CLP populations (145). These findings suggest a potential role for the CXCR4/CXCL12 axis in the CLP population by placing the CLP near CXCL12-abundant reticular cells, which have further been shown to provide CLPs with the pro-lymphoid cytokine IL7 (72, 126, 146). These observations highlight the potential for the Notch-CXCR4 pathway in lymphoid progenitor development and trafficking within the BM niche.

Lineage commitment of BM hematopoietic progenitors is a complex process involving coordination of cell fate determining transcriptional networks, which contribute to the heterogeneity of the progenitor pool (44, 86, 147). Given that Notch signaling is essential in the differentiation and maturation of thymic T cells, a direct functional role for Notch signaling in the early BM hematopoietic lineage decisions is a strong possibility (5, 25). Indeed, Dll4 expressed by vascular cells has been shown to suppress the myeloid transcriptional program in HSCs (104). Abrogation of Notch within hematopoietic progenitors leads to an altered myeloid differentiation program, with an increase in GMP production at the expense of MEPs and CMPs (19, 148). In the context of lymphoid progenitors, RNA sequencing has shown that Notch inhibits the myeloid program in both the thymic DN1a/b population and the CD62L^+^PSGL1^+^CCR9^+^ subset of LMPPs, which constitutes a putative TSP population (35, 58). Additionally, deletion of Dll4 in Ocn^+^ BM osteoblasts yields specific depletion of the T lineage primed Ly6D^-^ CLP population, hinting at a role for endosteal Notch signaling in influencing B cell vs T cell fates in BM CLPs (34, 73). Such a role has also been established in the thymus, as thymocyte Notch signaling inhibits expression of B-lineage specific factors EBF1 and Pax5 (80). Taken together, there are likely multiple distinct roles for the Notch pathway in the priming and generation of lymphoid progenitors in the BM, including activation of receptors involved in niche trafficking and repression of alternative lineage potential.

## Notch in BM Leukemic Niche

Notch signaling has been implicated in progression of various types of cancer, including Acute Lymphoblastic Leukemia (ALL) (149). Notch signaling plays a well-established role in T-cell acute lymphoblastic leukemia (T-ALL), which is a neoplasm of T-cell blasts accounting for 25% of adult ALL (150). Greater than 60% of patient samples contain mutations in the Notch pathway, with several gain of function mutations in the Notch1 gene, and inactivating mutations in negative regulators of Notch signaling, including FBW7 (151–158). Notch3, which is a Notch1 target gene, has also been shown to play a role in T-ALL (159–161). Mechanisms of Notch signaling in T-ALL oncogenesis include promotion of anabolic cell growth and chemoresistance, activation of the PI3K-AKT-mTOR pathway, and induction of genes involved in G1/S cell cycle progression (158, 162, 163). Notch has also been implicated in B cell leukemias. Hyperactive Notch1 and Notch2 have been shown to sustain B cell Chronic lymphocytic leukemia (B-CLL) (164–167). Conversely, all Notch receptors and the Notch target Hes5 have been shown to act as tumor suppressors in B cell ALL (B-ALL). Even so, Notch3 and Notch4 can prevent apoptosis of human B-ALL cells cultured on human stromal cells *ex vivo* (168–170).

**Figure 2:**
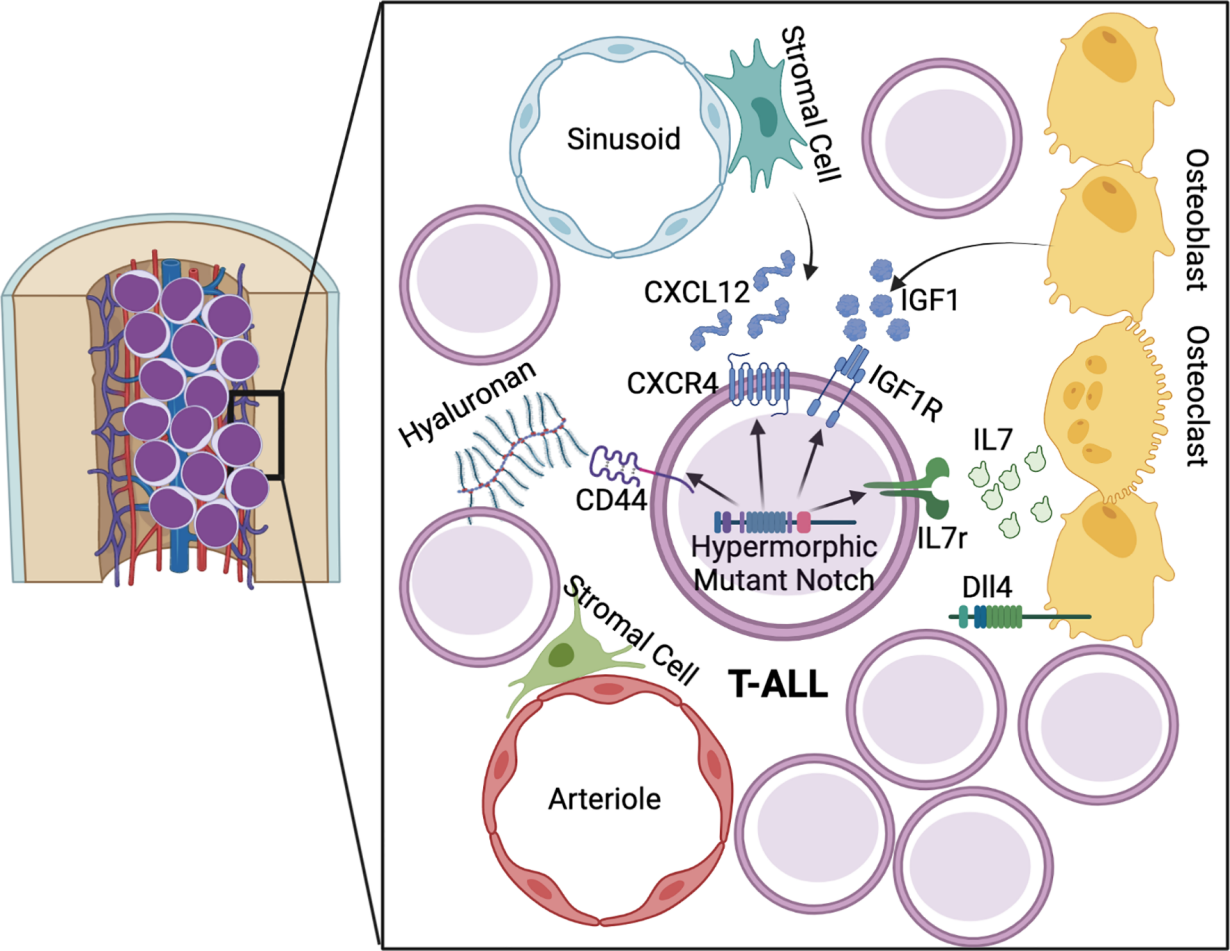
Notch driven mechanisms of T-ALL in the lymphoid niche. Hypermorphic Notch signaling promotes T-ALL progression and amplification of pathways involved in early BM lymphopoiesis. Growth factor signaling from IL7 and IGF1 are augmented *via* Notch driven expression of IL7r and IGF1R. CXCR4 and CD44 promote maintenance of LIC blasts in the BM microenvironment.

In the context of the BM microenvironment, leukemic cells have been shown to modulate the hematopoietic niche to form a pro-leukemic microenvironment at the expense of homeostatic hematopoiesis (**Figure 2**) (171). Interestingly, and unlike homeostatic lymphoid progenitors, Notch driven T-ALL cells are not maintained by a specific BM niche, but lead to remodeling of the endosteal niche and loss of osteoprogenitors (172). Such remodeling leads to perturbations in BM hematopoiesis, including reduced quiescence of HSCs and more severe leukemia progression (173). Additionally, a multitude of cell extrinsic signaling molecules have been implicated in the pathogenesis of T-ALL (174). The CXCL12/CXCR4 pathway is involved in homing of T-ALL cells to the bone marrow and in Leukemia Initiating Cell (LIC) activity (175). LICs propagate leukemia progression *via* their ability to both self-renew and produce clonal daughter blasts (176). Similarly to homeostatic HSPCs, T-ALL cells are subject to increased CXCR4 expression and activity downstream of Notch activation (144, 175, 177, 178). In human Chronic Lymphoid Leukemia (CLL) and Multiple Myeloma (MM), CXCR4 is also a direct transcriptional target of Notch1 (179, 180). Furthermore, CXCL12 receptor CXCR7 has been shown to be transcriptionally activated by Notch signaling in T-ALL and potentiates CXCR4 signaling and migration (181, 182). This pathway has become clinically relevant since CXCR4 inhibition has shown therapeutic potential in T-ALL (183). Specifically, direct CXCR4 antagonism prevents migration of CD4^+^/CD8^+^ leukemic cells from the thymus to the bone marrow in hypermorphic Notch3 transgenic mice (178). Furthermore, CXCR4 deletion in Notch1-induced T-ALL cells or CXCL12 deletion in endothelial, but not perivascular cells, limits T-ALL progression in mice through induction of cell death (177, 183). Thus, the CXCR4/CXCL12 axis, regulated in part by a hyperactive Notch pathway, is involved in the homing and progression of several leukemia subtypes in the BM niche.

IL7 signaling is critical for lymphopoiesis and, in the context of T-ALL, it plays a role in activation of the JAK/STAT5 and PI3K/Akt/mTOR pathways, with 10% of patient leukemia samples containing activating IL7r mutations (184–186). Stromal derived IL7 has been shown to activate the PI3K/Akt pathway, which is the dominant pathway mediating the proliferative and pro-survival signals downstream of IL7 in T-ALL cells (187–189). Notch1 activates IL7r transcription in human hematopoietic progenitors, as well as in murine leukemia cells (190–192). Additionally, in a human T cell leukemia cell line, IL7r has been shown to be directly co-regulated by Notch1 and RUNX1 (182). Thus, hyperactive Notch signaling contributes to the IL7 dependent proliferation of BM T-ALL cells. Another growth factor involved in BM lymphopoiesis is Insulin-like growth factor 1 (IGF1), which is released from osteoblasts, osteoclasts, and stromal cells in BM and is critical for bone growth (193, 194). Bone marrow levels of IGF1 decrease with age, resulting in increased myeloid bias of HSCs, while temporary IGF1 stimulation of murine hematopoietic progenitors *ex vivo* promotes lymphoid differentiation post-transplant in recipient mice (195). Notch1 has been shown to directly activate expression of Insulin-like growth factor 1 receptor (IGF1R) in T-ALL, which contributes to leukemia survival through the PI3K/Akt pathway (196, 197). IGF1R inhibition yields therapeutic benefits in several solid tumor types and leukemias (198). However, not all T-ALL cell lines are sensitive to IGF1R inhibition, with co-expression of surface IGF1R and tumor-suppressor PTEN indicating IGF1 dependence (196, 199). Interestingly, miR-233 has also been shown to be a Notch target which separately regulates IGF1R expression *via* targeting of the 3′ UTR and reduction of IGF1R protein levels in T-ALL (200). Taken together, Notch signaling in T-ALL allows for optimal signaling of BM derived growth factors, through regulation of their receptors.

Another important, though understudied, component of the BM niche is the extra-cellular matrix (ECM), which has been proposed to regulate both HSPCs and leukemia cells (201–204). The ECM is a vital component of structural and signaling mechanisms in all tissues and consists of collogens, proteoglycans, and glycoproteins (205–207). One protein involved in ECM binding is CD44, which is a cell adhesion molecule that binds to hyaluronan, fibronectin, collagen, E-selectin, and is involved in migration of fetal liver HSCs to the fetal BM (208–212). CD44 is also expressed on adult HSPCs and is involved in progenitor egress from the bone marrow and entry into the thymus (213, 214). Conversely, CD44 has been proposed to play a role in HSPC retention and quiescence, and contributes to apoptosis resistance in LICs (215). A potential mechanism of CD44 mediated chemoresistance in leukemia is through induction of drug efflux (216). In human T-ALL, CD44 has been proposed as a target of Notch1 and suggested to be required for BM engraftment of early leukemic cells (217). Additionally, CD44 can be used as a marker of LICs and is positively regulated by Notch signaling (218). In many tissues, additional ECM components have been shown to influence Notch activity, and there is cross-talk between ECM mediated signaling pathways and Notch (219). Microfibril Associated Glycoprotein-2 (MAGP-2) is found in elastic fibrils and has been shown to regulate Notch activity in COS cells and endothelial cells *via* binding to Notch1 Epidermal Growth Factor (EGF) repeats (220–222). The Cyr61, CTGF, and NOV, (CCN) family of ECM proteins influence osteogenesis and angiogenesis by binding to and enhancing Notch1 signaling (223–226). Additionally, Epidermal Growth Factor-Like Protein 7 (EGFL7), which is secreted by endothelial cells into the vascular microenvironment, regulates angiogenesis in part through antagonization of Notch signaling (227–230). These findings support the need for further exploration into the cross-talk and direct regulation between ECM components and the Notch pathway in T-ALL.

## Concluding Remarks

This review serves to highlight recent work which describes a pre-thymic niche in the BM where Notch signaling influences lymphoid, and specifically T cell, development. BM lymphoid progenitors receive Notch signals primarily in the osteoblastic niche, which also provides important signals involved in lymphoid development, including CXCL12, IL7, and SCF. Mechanisms of pre-thymic Notch signaling in BM lymphoid development include induction of molecules involved in bone egress and thymus migration. Ultimately a key outcome of this Notch signaling agenda is the early repression of the myeloid transcriptional program. However, while we know that Notch is active and functions in the BM, the downstream target genes of Notch activation in BM lymphopoiesis, particularly with respect to proliferation and survival, have yet to be fully established. We also examined the roles for aberrant Notch signaling in the BM migration, maintenance, and proliferation of T-ALL. Taken together, the works described here underscore the need for careful study of BM Notch signaling in lymphoid hematopoiesis.

## Author Contributions 

KS and KP wrote and edited this article. All authors contributed to the article and approved the submitted version.

## Funding

NIH/NHLBI R01HL134971, NIH/NHLBI T32HL007829-27.

## Conflict of Interest

The authors declare that the research was conducted in the absence of any commercial or financial relationships that could be construed as a potential conflict of interest.

## Publisher’s Note

All claims expressed in this article are solely those of the authors and do not necessarily represent those of their affiliated organizations, or those of the publisher, the editors and the reviewers. Any product that may be evaluated in this article, or claim that may be made by its manufacturer, is not guaranteed or endorsed by the publisher.
